# Factors influencing spousal support for women with perinatal depression in seeking formal assistance: a qualitative study

**DOI:** 10.3389/fpubh.2024.1493300

**Published:** 2024-11-15

**Authors:** Qinhan Zou, Yingzi Yang, Xianliang Liu, Tingting Wang, Ruizhe Chen, Xia Duan

**Affiliations:** ^1^School of Medicine, Tongji University, Shanghai, China; ^2^Department of Anesthesiology, Shanghai Ninth People’s Hospital Affiliated to Shanghai Jiao Tong University School of Medicine, Shanghai, China; ^3^Department of Health Care, Shanghai Health and Medical Center, Jiangsu, China; ^4^School of Nursing and Health Sciences, Hong Kong Metropolitan University, Homantin, Hong Kong SAR, China; ^5^Operation Room, The First Affiliated Hospital of WenZhou Medical University, Wen Zhou, China; ^6^Nursing Department, Shanghai First Maternity and Infant Hospital, School of Medicine, Tongji University, Shanghai, China

**Keywords:** perinatal depression, spouses, formal help, influencing factors, qualitative

## Abstract

**Objective:**

Seeking formal help can significantly improve the outcomes of perinatal depression (PND). However, currently, women with PND are not consistently seeking formal help. Research indicated that spouses played a crucial role in helping women recognize PND and encouraging them to seek formal help. This study aimed to explore the factors that prevent spouses from supporting women with PND in seeking formal help, based on the Knowledge-Attitude-Practice (KAP) theory.

**Methods:**

This is a qualitative study, utilizing semi-structured interviews to explore the factors that influence spouses to support women with PND to seek formal help. The interviews were conducted at a tertiary hospital in Shanghai, China from September 2023 to October 2023. Purposive sampling was used, and the sample size was determined by data saturation. Data analysis was conducted using Colaizzi’s seven-step method.

**Results:**

Twelve spouses had a mean age of 34.92 years (SD 5.81); *n* = 7 (58.33%) were new fathers. The influencing factors identified in this study can be explained by KAP theory, ultimately three major themes and six sub-themes emerged: (1) individual knowledge factors: lack of proper recognition of PND, (2) individual attitude factors: negative attitude toward PND screening and treatment and (3) service provider factors: imbalance between supply and demand for perinatal mental health services.

**Conclusion:**

Spouses who lacked supportive behavior were influenced by individual factors, including knowledge factors and attitude factors, as well as service provider factors. These identified factors can guide future research and the development of interventions to improve perinatal mental health services and encourage family support in seeking formal help.

## 1 Introduction

Perinatal depression (PND) is characterized by symptoms such as low mood, loss of interest, fatigue, appetite changes, sleep disturbances, feelings of guilt and suicidal thoughts in women, including prenatal depression and postpartum depression (PPD), with prevalence rates observed in China ranging from 15 to 20% among women during pregnancy and up to 1 year after childbirth (1, 2). PND not only causes intense sadness and anxiety in women but also endangers their health and that of their babies, strains family relationships, and can lead to self-harm, harm to their babies, or depression in their spouses (3–5). The most serious consequence of PND is suicide. Most suicides occurred within a year of childbirth, and PPD accounted for 20% of suicides (6). PND has cascading and lasting effects on mothers and their families, making it a global health issue (7).

In order to prevent these adverse consequences, it is necessary to encourage women to seek formal help. Seeking formal help refers to seeking help from healthcare professionals and medical staff for professional support, advice, and related treatment (8). Women with PND who seek formal help often resolve their psychological problems, reduce their risk of PPD, adjust to motherhood more quickly, care better for their babies, and are more likely to seek help if they experience PND again (9, 10). Despite the effectiveness of formal help in improving PND symptoms, only 17–25% of women with PND actually receive it (11). A study in Hunan, China, found that only 9.3% of women with PND sought formal help as their first choice after screening (12).

At present, there is evidence that women’s support networks, especially their partners, may play a facilitating role in the process of seeking formal help, and the decision to seek formal help is often not made by women alone. Spouses are a crucial source of social support for women, and they play a key role in identifying PND and influencing women’s decisions to seek help (13–15). Spouses impact the decision-making process, offer encouragement, and may take the initiative to seek help on behalf of the women (15, 16). Women who are encouraged by their spouse to seek formal help during the perinatal period are more likely to seek formal help when dealing with psychological issues (17). Given the importance of seeking formal help in managing women’s PND, most women currently do not seek help. Therefore, it is essential to explore why some spouses do not support women with PND in seeking formal help and to develop interventions to promote supportive behaviors. Factors such as lower education, higher stigma, and adherence to traditional cultures have been shown to discourage spouses from seeking formal help (18–20). These studies only found factors that influence spouses not to seek formal help, and could not explain why spouses do not support their wives in seeking formal help. Utilizing qualitative research can help identify the influencing factors based on spouses’ perceptions and related experiences of seeking formal help.

The Knowledge-Attitude-Practice (KAP) theory, first proposed by Hochbaum in the mid-19th century for evaluating family planning, suggested that behavior was influenced by an individual’s attitudes and knowledge (21). Studies have used KAP to explore factors that influence health behaviors (21, 22), but there is a lack of research to understand that spouses who do not support seeking formal help. Therefore, this study aimed to explore factors that influence why spouses do not support women with PND seeking formal help during the perinatal period. The study used the KAP theory to explain how knowledge, attitudes, and other factors can influence a spouse’s unsupportive behavior.

## 2 Methods

### 2.1 Study design

A descriptive qualitative research method (23) informed the design of this study, and semi-structured interviews were used to investigate the factors influencing spouses’ lack of support for women with PND in seeking formal help. Qualitative description study is a direct description of an event or experience that tends to elicit direct or essential answers to questions of concern to practitioners or policy makers (24). The study adhered to the Consolidated Criteria for Reporting Qualitative Research (COREQ) (25).

### 2.2 Participants and recruitment

Participants were recruited from the First Maternity and Infant Hospital Affiliated to Tongji University in Shanghai, China, using a purposive sampling method from September 2023 to October 2023. Inclusion criteria of participants who were: (1) they were spouses of women with PND, as assessed by the Edinburgh Postpartum Depression Scale (EPDS) (26); (2) aged 18 years or older; (3) able to read, write, and speak Chinese; and (4) willing to participate. Exclusion criteria included serious mental or psychiatric disorders (e.g., schizophrenia and bipolar disorder). We used the maximum variation sampling method, and the sampling was based on a maximum variation in participants’ characteristics (e.g., age, education and parental experience) (27). After completing interviews with 12 spouses, we confirmed that the data was saturated and no new topics emerged. The potential participants were contacted via telephone. Prior to the interview, the researcher provided a detailed explanation of the purpose and content of this study. All participants agreed to take part in the study and signed an informed consent. In appreciation of their valuable time and effort, each participant received two burp cloths.

### 2.3 Data collection

Data were collected through semi-structured face-to-face interviews conducted in the maternity and infant clinic. The interviews were conducted with spouses only, in a relaxed environment. The semi-structured interview guide was developed through a comprehensive process including literature review, input from a team of experts, and the researcher’s clinical experience in the field (two researchers, XD and XL held Ph.D., two (RC and YY) held master’s degrees, and two (QZ and TW) were master’s students in nursing). The semi-structured interview guide has been tested and revised in pilot interviews with three participants (Table 1).

**Table 1:** Semi-structured interview guide.

No.	Questions
1	Do you know what perinatal depression is? Could you tell me more about it?
2	How would you describe your wife’s emotional problems during the perinatal period?
3	Have you and your wife been screened for perinatal depression? Could you tell me what you think about this screening?
4	What’s your opinion on seeking formal help for perinatal depression?
5	Have you encouraged support your wife with perinatal depression to seek formal help? Why? Would you elaborate on this?
6	What do you think of the current treatment of depression? If you have a psychological problem, how do you solve it?
7	Have you and your wife received any support from maternal and child health care staff recently? What specific help or support do you require?

The researchers all have extensive experience in qualitative research and have received qualitative research training for this study. After the researcher introduced the purpose and significance of the study to participants and asked for their consent. The interview was conducted in a quiet room of the maternity and infant clinic. To assess the validity of the interviews questions, we conducted pre-interviews with three spouses. During the interview, the researcher flexibly adjusted the order and method of interview questions according to the interview outlines and the actual of participants. Although the interview questions were semi-structured, participants were free to express new opinions during the interview. Interviews were audio-recorded, and the participants’ body movements and expressions were also observed and recorded. To protect privacy, participants were coded from A1 to A12. To ensure accuracy, transcripts were confirmed with participants at the end of interviews.

### 2.4 Ethical considerations

This study conformed to the requirements of Helsinki Declaration and received ethical approval from the First Maternity and Infant Hospital Affiliated to Tongji University ethics committee (No. KS21273). Participation was voluntary, with informed consent obtained from all participants. Participants were informed about the study’s purpose, procedures, benefits, and risks. Their identities were coded to ensure privacy, and data were used solely for this study with their consent. Participants could withdraw from the study at any time.

### 2.5 Data analysis

Each interview lasted between 30 and 40 min. Interviews were transcribed within 24 h and analyzed using NVivo 12.0 software, and non-verbal actions were merged with the written transcripts. Two researchers independently analyzed the data using Colaizzi’s seven-step method (28) combined with the KAP theoretical framework. The deductive data analysis method utilized in this qualitative study, under the guidance of the KAP theoretical model, intricately investigated the factors contributing to unsupportive behaviors in spouses. The analysis process was shown in Table 2. When analyzing the data, the researchers avoided personal values and biases and discussed and revised their findings with the team members.

**Table 2:** Colaizzi’s seven-step method analysis process combined with KAP theoretical framework.

Step	Data analysis process
1	The researchers read all interview data several times and had a preliminary understanding of the interview data.
2	The researcher reread and marked meaningful statements consistent with the research question.
3	Meaningful statements were summarized, refined and coded.
4	The researchers summarized meaningful statements, found common concepts and formed themes and sub-themes. The themes and sub-themes were consistent with the dimensions of KAP theory.
5	Themes and sub-themes were linked to the participants and described in detail, with an explanation of the participants’ actual unsupportive behavior.
6	The researchers described the basic structure of this phenomenon.
7	The final analysis results were returned to the participants for verification. Researchers avoided personal biases and discussed findings with team members.

### 2.6 Rigor

Four criteria were used to ensure the rigor of the research (29): credibility, reliability, confirmability and transferability. To ensure credibility, researcher triangulation was incorporated into the analysis strategy. Reliability was ensured by providing the data to an experienced external researcher for an independent check. Confirmability was ensured by using audit tests to verify whether the authors were biased against the findings. For transferability, we clearly described the research background, sampling methods, and data collection and analysis methods.

## 3 Results

In this study, nine women had mild depression and three had severe depression. Twelve spouses participated, coded from A1 to A12, with an average age of 34.92 ± 5.81 years. Most participants had a vocational high school education. Seven spouses were new fathers and five spouses were experienced fathers. Four spouses knew about PND, and the other eight did not. The general characteristics of the participants are shown in Table 3.

**Table 3:** Characteristics of 12 spouses.

Participants	Coded as	Age	Education	New^2^/experienced^3^ father	Degree of PND in woman^1^	Knowledge of PND	PND screening intention	Attitude toward depression treatment	Intention to accept referrals^4^	Stage of pregnancy in woman^1^
Participant 1	A1	32	University	New	Mild	No	Refuse	Disagree	Refuse	Postpartum
Participant 2	A2	29	Vocational high school	New	Mild	No	Refuse	Disagree	Refuse	Antenatal
Participant 3	A3	40	Vocational high school	Experienced	Severe	Yes	Refuse	Doubting	Refuse	Postpartum
Participant 4	A4	48	Vocational high school	Experienced	Severe	Yes	Accept	Doubting	Neutral	Antenatal
Participant 5	A5	32	University	Experienced	Mild	No	Refuse	Agree	Refuse	Antenatal
Participant 6	A6	28	University	New	Mild	No	Refuse	Disagree	Refuse	Antenatal
Participant 7	A7	41	University	Experienced	Mild	No	Refuse	Doubting	Refuse	Postpartum
Participant 8	A8	31	Master degree candidate	New	Mild	No	Accept	Agree	Refuse	Postpartum
Participant 9	A9	34	Junior high school	Experienced	Mild	Yes	Refuse	Disagree	Refuse	Antenatal
Participant 10	A10	31	University	New	Severe	No	Refuse	Agree	Refuse	Antenatal
Participant 11	A11	37	Vocational high school	New	Mild	Yes	Refuse	Doubting	Neutral	Postpartum
Participant 12	A12	36	Vocational high school	New	Mild	No	Refuse	Disagree	Agree	Antenatal

1 At the time of PND.

2 A new father was defined as someone who has recently had a child.

3 An experienced father was defined as having two or more children.

4 A referral was defined as an obstetrician referring a woman with psychological problems to the designated psychiatric hospital.

**Figure 1:**
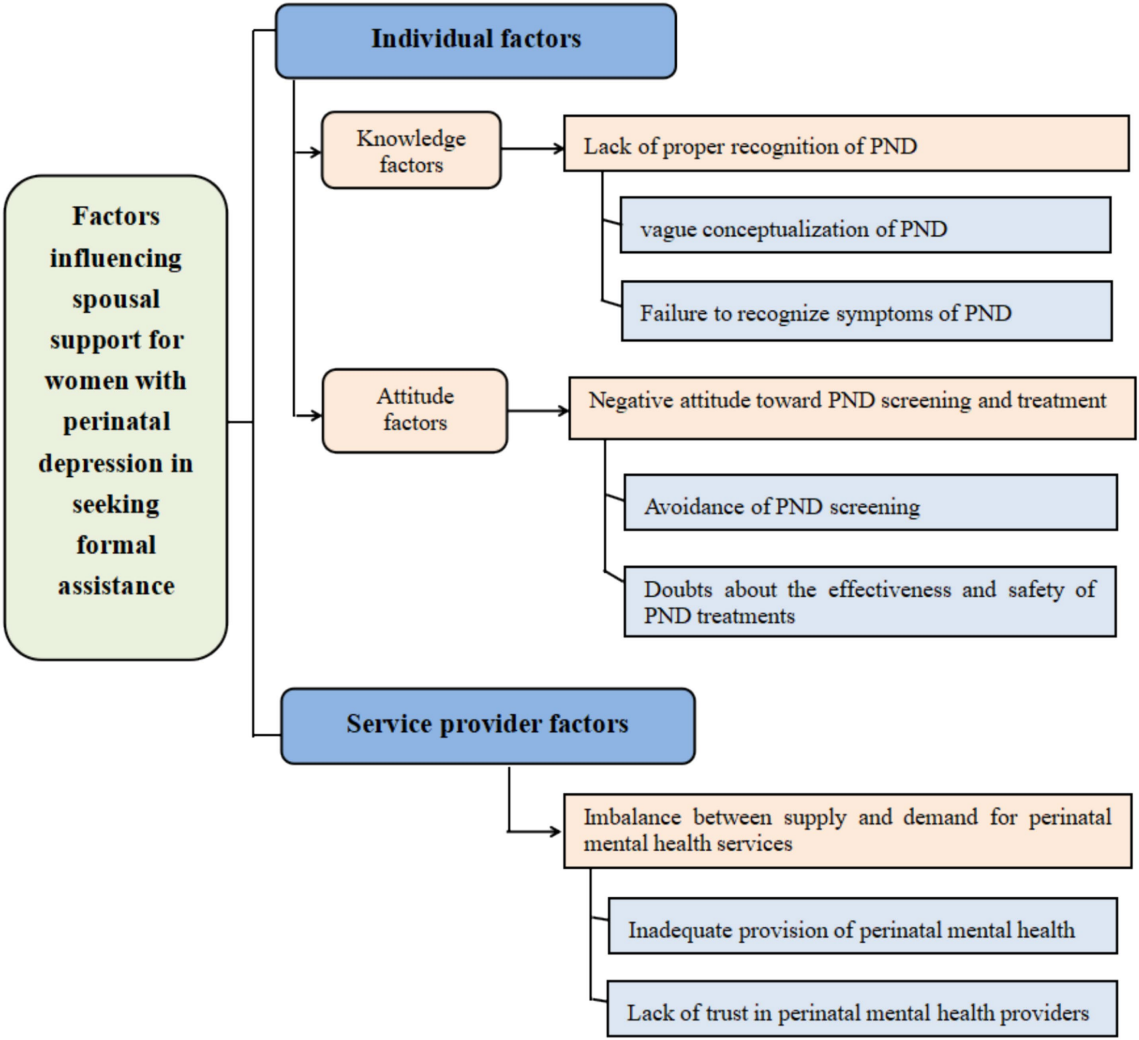
Themes and sub-themes.

The identified themes and six sub-themes were shown in Figure 1 and detailed below: (1) individual knowledge factors: lack of proper recognition of PND, (2) individual attitude factors: negative attitude toward PND screening and treatment and (3) service provider factors: imbalance between supply and demand for perinatal mental health services.

**Figure 2:**
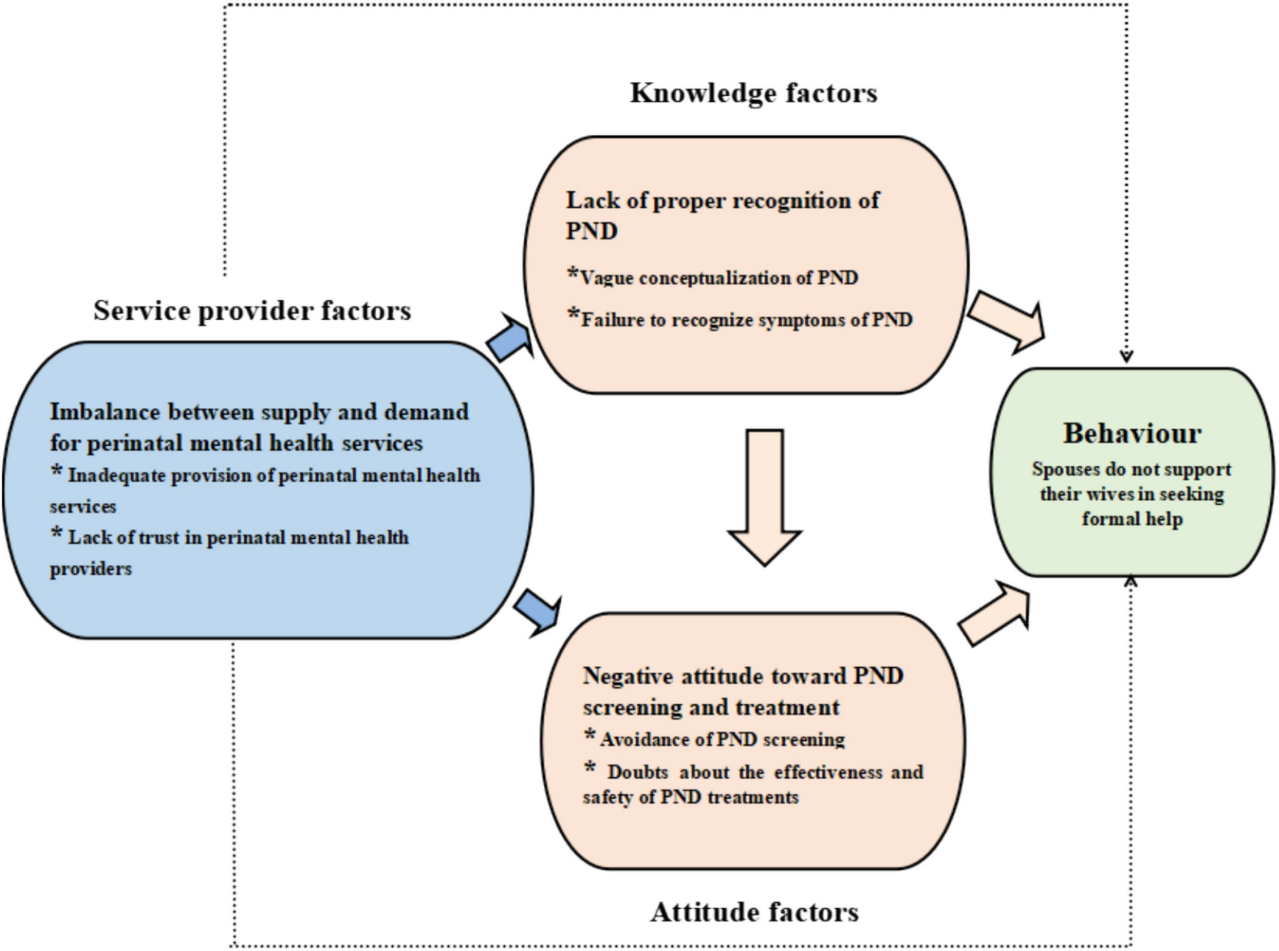
KAP theoretical model of factors influencing spouses no supporting for women with PND to seek formal help.

The data analysis process in this study, guided by the KAP theoretical model, facilitated a comprehensive examination of the factors influencing unsupportive behaviors in spouses. It elucidated how these factors contribute to spouses’ failure to recognize symptoms of PND and their reluctance to undergo screening and treatment. The study revealed that knowledge and attitude factors, alongside service provider factors such as the inadequate provision of perinatal mental health services and a lack of recognition of the professionalism of maternal and child health (MCH) care staff in mental health services, are significant contributors to unsupportive behaviors in spouses (please refer to Figure 2).

### 3.1 Theme 1-individual knowledge factors: lack of proper recognition of PND

This theme addressed spouses’ knowledge and recognition of PND symptoms, categorizing them as individual knowledge factors. The sub-themes were: vague conceptualization of PND, and failure to recognize symptoms of PND.

#### 3.1.1 Sub-theme-vague conceptualization of PND

Only four spouses had heard of PND, and three were experienced fathers. They learned about PND through MCH care staff and friends. Experienced fathers were more concerned about their wives’ mental health but still had a vague understanding of PND.

“*My wife is currently pregnant with our second child. As a result of the parenting experience we gained raising our first child, I’m more concerned about my wife’s mental health and have bought books about perinatal depression. However, I can only get a simple understanding of perinatal depression through books*” (**A5**).

“*When my wife was pregnant with our first child, MCH care staff explained what perinatal depression was and my friends talked to me about it. But I still do not know about it”* (**A3**).

When spouses were asked if they knew about PND. They hesitated for a moment before answering. We found that new fathers were unaware of PND and equated it with postpartum depression (PPD).

*“What is perinatal depression? I’ve never heard of it. This is the first time I have heard about perinatal depression. Through your introduction, I learned that women can suffer from prenatal depression”* (**A10**).

“*I know perinatal depression, that’s postpartum depression, right? There have been TV reports of women with postpartum depression committing suicide*” (**A12**).

#### 3.1.2 Sub-theme-failure to recognize symptoms of PND

Spouses failed to recognize wives’ psychological problems and attributed the symptoms of PND to the stress of parenthood. When they talked about the stress of parenthood, they got upset and sighed more.

*“We are new parents and life is full of challenges. We are trying to become ideal parents, but unmet expectations and underlying feelings of inadequacy have caused us anxiety and depression”* (**A8**).

They believed that the wife’s depressive symptoms were a normal reaction to pregnancy, leading to the wife’s distress being minimized or ignored.

*“I did not know that she might have been depressed for three months. In the third trimester of her pregnancy, my wife complained frequently of headaches, lack of sleep and weakness. My first reaction was that her belly was too big, her body was heavy, and she was not resting well”* (**A12**).

Spouses also perceived perinatal depressive symptoms as anxiety about physical appearance. Therefore, in the interview, the spouse expressed helplessness about the wife’s appearance anxiety and asked for help dissuading the wife.

*“My wife gained [significant weight] after her pregnancy. She thinks her body is bloated. She hugged me and cried and said she was afraid to look in the mirror. I think she is too conscious of her physical appearance”* (**A10**).

### 3.2 Theme 2-individual attitude factors: negative attitude toward PND screening and treatment

The second theme reflected spouses’ attitudes toward PND screening and treatment, forming an individual attitude factors. The sub-themes were: avoidance of PND screening, and Doubts about the effectiveness and safety of PND treatments.

#### 3.2.1 Sub-theme-avoidance of PND screening

Ten spouses refused to take up the PND screening service offered by MCH care staff. They noted that perinatal care services should not include PND screening and that only women with psychological problems should be screened.

*“I think my wife’s emotional state is normal. We do not need perinatal depression screening”* (**A6**).

This study also found that spouses were concerned that screening for PND would be a waste of time and increase costs.

*“My wife and I work and look after our children at the same time, so we do not have any extra time for perinatal depression screening”* (**A7**).

“*Screening for psychological problems is known to be expensive. Our income is low and we do not want to pay for unnecessary screening”* (**A2**).

#### 3.2.2 Sub-theme-doubts about the effectiveness and safety of PND treatments

When spouses were asked about their views on psychotherapy, some spouses disagreed with its effectiveness. These spouses described they had no prior psychological issues. They stated that if either they or their wives experienced depression, they would manage it themselves.

*“I believe that depression occurs in people who are less able to cope with stress and that psychotherapy does not improve people’s ability to cope with stress. If I feel depressed, I will choose to talk to friends or family members or do something that relaxes me”* (**A9**).

*“The stresses of today’s society (work problems, financial problems, relationship problems, etc.) cause depression in everyone. Most people choose self-regulation to cope with depression, and people with severe depression choose psychotherapy. But psychotherapy does not always work. For example, [my family member] has psychotherapy twice a week. He has been in therapy for a year, but his depressive symptoms have not improved”* (**A12**).

During a discussion about the necessity of taking antidepressants, four spouses expressed their concerns to the interviewer regarding whether the side effects of the medication might affect the health of their unborn children. They felt guilty for not supporting their wives in seeking treatment, even though they were equally worried about their wives’ mental health.

“*I have learnt that people with severe depression need to be treated with medication. If my wife is taking medication for depression, I am worried that the side effects of the medication will affect the child’s intelligence”* (**A4**).

### 3.3 Theme 3-service provider factors: imbalance between supply and demand for perinatal mental health services

This theme highlighted issues of MCH care staff in the provision of perinatal mental health and the spouse’s view of the role of MCH care staff in perinatal mental health services, which were classified as service provider factors. The sub-themes were: inadequate provision of perinatal mental health services and lack of trust in perinatal mental health providers.

#### 3.3.1 Sub-theme-inadequate provision of perinatal mental health services

The researcher then asked the spouses if they had read the books on the shelf next to the nurses’ station. Six spouses said they were not interested in reading them, and another six said they would not read them even if they were interested. They were embarrassed by the act of asking for the books and at the same time MCH care staff did not tell them that the books could be read for free.

*“I noticed a bookshelf next to the nurses’ station with books on pregnancy and childbirth. Although I’d passed the bookshelf many times, I had not looked through the books. I thought it would be embarrassing to ask the nurses for them. I would have appreciated it if the nurses had offered to give me some books. I will read these books at my leisure.”*(**A4**).

Almost all spouses said that MCH care staff did not mention psychological problems when they accompanied their wives for antenatal check-ups.

*“Every time we go for antenatal check-ups, the doctors and nurses only focus on the physical of the mother and baby”* (**A8**).

#### 3.3.2 Sub-theme-lack of trust in perinatal mental health providers

Our study found that spouses thought that psychological problems should be treated by psychologists, and MCH care staff should not diagnose psychological problems and provide referral services. For women with severe depression, obstetricians should recommend visits to designated psychological clinics or hospitals. Additionally, the obstetrician’s team should coordinate with psychologists to ensure proper treatment for depressed women.

*“I think psychological professionals should treat psychological problems and MCH care staff should treat physical problems of mothers and babies.* If my wife has a mental health issue, we will go to a psychiatric hospital or the psychiatric department of a general hospital for treatment. We will also cooperate with the follow-up services provided by the mental health staff*”* (**A5**).

Three spouses were neutral. They summarized their acceptance of the perinatal mental health services provided by MCH care staff in terms of “maybe” or “probably.”

*“Women’s psychological problems are also part of the work of MCH care staff. I maybe follow their advice. For example, they advised me to go to designated psychological clinics or hospitals”* (**A11**).

“*Before deciding whether to accept the referral, I will record the address of the designated psychological hospital as provided by my obstetrician and conduct an online search to gather additional information. Furthermore, I would be grateful if the obstetrician could supply me with a contact number for further consultation*” (**A4**).

Only one spouse expressed trust in the MCH care staff. This spouse said that if the MCH care staff had diagnosed his wife with psychological problems, he would follow the advice and accepted the referral.

*“The obstetrician was professional, the nurses were friendly and their answers to questions were carefully and accurately. My wife and I know nothing about PND. Currently, MCH care staff are our closest contact and only through them can we recognize psychological issues. The psychotherapists and hospitals they recommend for treatment are reliable”* (**A12**).

## 4 Discussion

By exploring the influencing factors of spouses not supporting women with PND to seek formal help, our study found three influencing factors and three themes. This study clarified that promoting the ability of MCH care staff to provide mental health services and optimizing perinatal mental health services are important factors in promoting spousal support for women with PND seeking help. Our findings align with existing literature (30, 31) regarding spouses’ limited recognition of PND and their skepticism toward the role of MCH care staff in addressing perinatal mental health issues.

Through in-depth interviews with spouses, we found that spouses failed to recognize PND, some of them equated PND with postpartum depression (PPD). Lack of knowledge about PND affects spouses’ ability to recognize, cope with and manage PND, which is one of the biggest barriers for them to seek formal help (31). During the interviews, experienced fathers demonstrated a greater understanding of PND compared to new fathers, who were often younger and less experienced in parenting. New fathers primarily focused on striving to be good parents but generally lacked knowledge about PND. As a result, they often overlooked their wives’ mental health needs. Many new fathers mistakenly viewed prenatal depression as a normal response to pregnancy, even when their wives reported symptoms such as headaches and sleep problems. We also found that new fathers misinterpret their wives’ depression as appearance anxiety, leaving them helpless in the face of their crying wives. This study’s finding that spouses normalize their wives’ depressive symptoms is consistent with findings of Bitew et al. (32). Our findings emphasize that MCH care staff should not only pay more attention to perinatal mental health education and help spouses understand of PND. There is also a need to focus on the parenting pressures of new fathers and provide targeted support to direct their attention to mental health.

In terms of attitude factors, spouses avoidance of PND screening in this study seriously hindered the recognition of PND in women. PND screening is crucial for helping MCH care staff and couples identify depression and facilitate couples seeking formal help (33). Without screening, many women with PND neither receive treatment nor seek help promptly. Participation in PND screening improved referrals for mental health support and increased use of mental health services (34, 35). Some spouses, especially new fathers, considered their wives’ emotions ‘normal’, downplaying the importance of screening and discouraged their wives from attending PND screening (36). It is recommended to inform spouses of the need and benefits of early PND screening, including timely detection of PND and savings medical costs. Previous research has reported on the screening experiences of women who perceived screening for emotional problems by healthcare professionals (HCPs) as a ‘tick-box’ approach rather than real care (37), and the shorter screening process prevents women from fully disclosing their condition (38). Therefore, when implementing PND screening, the screening information should be clear and detailed, and the feelings of the participants should be fully considered.

Another attitude factor that emerged was that the effectiveness and safety of treatments for PND was questioned by spouses. Formal treatment has been shown to be effective in treating PND. Women who have received formal help are more likely to believe in its benefits and recommend it to others in need (4, 9, 10). Therefore, MCH care staff can educate spouses about the effectiveness of formal treatments by sharing successful cases of PND recovery. A significant challenge for women with PND and their spouses is deciding whether to start treatment during pregnancy due to fears that depression treatment might harm the fetus (6). In our interviews, spouses with lower education (vocational high school and blew) were more likely to doubt the safety of PND treatments. Treatments for PND include both non-pharmacological options, such as psychotherapy, and pharmacological options, tailored to the severity of the woman’s depression. Many psychiatric medications now have minimal side effects during pregnancy and breastfeeding, making them safer for the mother’s health and the child’s development compared to untreated depression (4). Spouses with lower education only considered the side effects of the drugs and did not support the wife in receiving treatment, without realizing that the consequences of refusing treatment could be more serious than the potential risks of treatment. Therefore, MCH care staff need to improve the awareness of PND treatment among spouses and change the inherent perception of depression treatment among spouses with lower education.

This study also reported that service provider factors influenced expressed a lack of trust in the role of MCH care staff in the provision of perinatal mental health services. Through qualitative interviews conducted in this study, three distinct attitudes among spouses regarding referrals made by MCH care staff were identified. Firstly, spouses who refuse referral state that they will only accept treatment advice and follow-up from psychiatrists. Secondly, the neutral spouse expected the obstetricians to provide both the address and a contact number for consultations at the psychiatric hospital. Finally, spouses who have established a trusting relationship with obstetricians are willing to seek treatment at the designated psychiatric hospitals. Bennett et al. reported that pregnant women who discussed depression with their obstetrician during antenatal care were more likely to seek help from their obstetricians if they had depression (39). Similarly, women were more likely to disclose depressive symptoms when there was a trusting and respectful therapeutic relationship with HCPs (40). Our findings align with the findings of these studies. MCH care staff should focus on building therapeutic relationships with couples based on mutual trust. Spouses reported that during the perinatal period, no MCH care staff offered PND screening or mental health counseling services. When obstetricians suspect a woman has a psychological issue, they typically recommend referring her to a psychiatric hospital. Treatment for depression and subsequent monitoring are managed by mental health professionals. Currently, there exists a significant communication gap between psychological professionals and MCH care staff, as the exchange of patient information predominantly relies on the use of medical files rather than direct dialogue. This lack of direct communication hinders timely monitoring and referral of patients with PND (41). For effective monitoring and timely referral, it is essential to strengthen communication and collaboration between MCH care staff and psychiatric professionals. Lack of communication time and feeling unsupported were major barriers to seeking formal help, while the professionalism of health professionals promoted trust building and seeking help (30, 42). To improve perinatal psychological services, it is essential to strengthen the capabilities of MCH staff. This can be achieved by integrating screening and counseling for couples, while also encouraging direct communication between psychological professionals and MCH personnel.

## 5 Strengths and limitations

This study explored the factors influencing women with PND not seeking formal help from the perspective of their spouses, contributing new insights to the existing literature. In qualitative studies, the data obtained represent only narrative interviews from a sample of participants, which means that they are limited in their representativeness. In addition, being limited to one study location means that this study lacks a broader perspective of the spouses. Future research should consider a more diverse and comprehensive sample to validate and expand upon these findings.

## 6 Conclusion

The present study has successfully delineated key factors shaping unsupportive behaviors among spouses, hindering women with PND from seeking formal assistance in the perinatal phase, and elucidating how these factors intertwine within the KAP theoretical framework. These insights can guide MCH care staff in enhancing support for women with PND and their partners, fostering familial understanding and paving the way for future research endeavors in this domain.

## Data Availability

The original contributions presented in the study are included in the article/supplementary material, further inquiries can be directed to the corresponding author.
